# Looking beyond discharge: clinical variables at trauma admission predict long term survival in the older severely injured patient

**DOI:** 10.1186/1749-7922-9-10

**Published:** 2014-01-23

**Authors:** Miklosh Bala, Jeffry L Kashuk, Dafna Willner, Dima Kaluzhni, Tali Bdolah-Abram, Gidon Almogy

**Affiliations:** 1 Department of Surgery and Shock Trauma Unit, Hadassah-Hebrew University Medical Center, Jerusalem, Israel; 2 Director of Surgical Research and Academic Development, EM Care Acute Care Surgery, Dallas, Texas, USA; 3 Department of Anesthesiology and Intensive Care Unit, Hadassah-Hebrew University Medical Center, Jerusalem, Israel; 4 Department of Social Medicine, Hadassah-Hebrew University Medical Center, Jerusalem, Israel

## Abstract

**Background:**

Long term follow up is difficult to obtain in most trauma settings, these data are essential for assessing outcomes in the older (≥60) patient. We hypothesized that clinical data obtained during initial hospital stay could accurately predict long term survival.

**Study design:**

Using our trauma registry and hospital database, we reviewed all trauma admissions (age ≥60, ISS > 15) to our Level 1 center over the most recent 7 years. Mechanism of injury, co-morbidities, ICU admission, and ultimate disposition were assessed for 2-7 years post-discharge. Primary outcome was defined as long term survival to the end of the last year of the study.

**Results:**

Of 342 patients discharged following initial admission, mean age was 76.2 ± 9.7, and ISS was 21.5 ± 6.9. 119 patients (34.8%) died (mean follow up 18.8 months; range 1.1-66.2 months). For 233 survivors, mean follow-up was 50.2 months (range 24.8-83.8 months). Univariate analysis disclosed post-discharge mortality was associated with age (80.1 ± 9.64 vs. 74.2 ± 9.07), mean number of co-morbidities (1.6 ± 1.1 vs. 1.0 ± 1.2), fall as a mechanism, lower GCS upon arrival (11.85 ± 4.21 vs. 13.73 ± 2.89), intubation at the scene and discharge to an assisted living facility (p < 0.001 for all). Cox regression analysis hazard ratio showed that independent predictors of mortality on long term follow-up included: older age, fall as mechanism, lower GCS at admission and discharge to assisted living facility (all = p < 0.0001).

**Conclusions:**

Nearly two-thirds of patients ≥60 who were severely injured survived >4 years following discharge; furthermore, admission data, including younger age, injury mechanism other than falls, higher GCS and home discharge predicted a favorable long term outcome. These findings suggest that common clinical data at initial admission can predict long term survival in the older trauma patient.

## Introduction

The population of the western world is simultaneously aging and living longer. In Israel, the rate of increase of the elderly population is expected to be 2.5 times that of the general population [1]. Furthermore, as is the case in Japan, Australia, and Sweden, Israel has the highest life expectancy for males at birth in the world (79 years) [2]. Along with the prolonged life expectancy, seniors also have an improved quality of life, with increased strength and vigor, resulting in greater physical activity and mobility. Accordingly, all of these factors have resulted in a noticeable increase in the number of seniors with severe traumatic injuries presenting to our trauma center with falls and motor vehicle crashes as the predominant mechanisms of injury [3-5].

The care and treatment of elderly trauma patients is particularly challenging to the trauma surgeon, as advanced age, extensive past medical history, and poor physiologic reserve are well-recognized risk factors for adverse outcomes following trauma [6,7]. Attempts to better characterize physiologic deficiencies in the elderly have recently been assessed via calculation of frailty indices in order to predict 6-month postoperative mortality and post-discharge institutionalization [8]. Despite increasing recognition of the unique challenges of the senior population to trauma care, little information is currently available regarding specific factors that predict morbidity and mortality in this group, including an improved understanding of long term outcome following discharge [9,10]. Others have shown that the outcome of elderly trauma patients hospitalized in major trauma centers is better than can be predicted based on current indices and therefore, aggressive treatment may improve their chances of regaining their pre-injury status. Lastly, not only in the senior population but in all trauma patients, increasing costs of care have led to careful considerations of resource allocation and improved recognition of scenarios where care may be futile [10].

Based upon all of the above factors, our primary objective in the current study was to describe and define the long term outcome of elderly patients following severe trauma in our Israeli level 1 regional trauma center over the most recent 7 year time frame. Our secondary objective was to identify predictors of long term survival in this population.

## Methods

We searched our trauma data base for all trauma patients ≥60 years of age who presented to Trauma Unit of Hadassah University Medical Center, Ein Kerem campus, Jerusalem, the regional Level I Trauma Center, with an ISS of ≥16 between January 2006 and December 2010. Discharged patients were followed after discharge either home or to institutional placement for the duration of the study time frame or until mortality. Long term follow up was recorded on survivors discharged from hospital following admission from January 2006. Exclusion criteria included patients who were pronounced dead upon arrival and patients who were transferred from other acute care hospitals.

All charts were retrospectively reviewed for demographics (age, gender, pre-existing co-morbidities, pre-existing anticoagulation medications, mechanism of injury, ISS, head abbreviated injury score [AIS], GCS at scene and upon presentation to the ED, intubation at scene or in ED, injured body regions, admission serum creatinine and INR, intensive care unit length of stay (ICU LOS), hospital LOS, surgical interventions, complications (infectious and non-infectious), and in-hospital mortality.

Any mortality within 30 days of injury was considered an in-hospital death regardless of patient location at the time of death. Time of death was extracted from the medical records which are updated regularly by the Israeli Governmental Ministry of Internal Affairs registry. Outcome variables were mortality and discharge placement. Discharge placement was defined as the patient destination after acute care in the trauma center, being home, rehabilitation center, assisted-living facility (ALF) (defined as lower level of dependence requiring professional support), or transfer to another acute care hospital. Co-morbidities were defined as noted in Table 1. The absolute number of co-morbidities was calculated for patients with more than one listed illness.

**Table 1:** Definition of co-morbidities identified in the study population

	
Cardiac disease	Known history of ischemic heart disease, previous cardiac interventions
Malignancy	Currently under oncological follow up or treatment for active oncological disease
Diabetes mellitus	Patient requiring insulin or oral hypoglycemic therapy
Neurological disease	History of cerebro-vascular accident, severe parkinsonism and/ or antiepileptic therapy
Dementia	Any case with established diagnosis of dementia
Hypertension	History of hypertension requiring medication
Chronic anticoagulation	Patients currently on anticoagulation (LMWH or Warfarin), and /or antiplatelet therapy (excluding aspirin)
Chronic renal failure	History of preexisting renal insufficiency on admission
Chronic obstructive pulmonary disease	Ongoing treatment for COPD

### Statistical analysis

For quantitative variables, data is presented as mean and standard deviation (SD). The Chi-square test as well as the Fisher’s exact test was used to test the association between two qualitative variables. The Chi-square test for trends was used for qualitative ordinal variables. The Student’s T test was used to compare quantitative variables between the two groups. Univariate survival analysis was performed by Kaplan-Meier (K-M) methodology with significance of the difference between survival curves determined by the log-rank test. Variables which were significant in the K-M analysis, were entered into a stepwise, (forward, likelihood ratio) Cox regression model. A logistic regression model was used to define predictors of death during the follow up period.

All tests applied were two-tailed, with p value of 0.05 or less considered statistically significant. Statistical analysis was performed using IBM SPSS Statistics (IBM Corp. Released 2011. IBM SPSS Statistics for Windows, Version 20.0. Armonk, NY: IBM Corp.)

## Results

### Patient population

416 patients ≥60 years of age with an ISS ≥16 met inclusion criteria with complete data, and were identified who presented to our trauma unit during the study period. Mean age was 76.9 ± 9.6 years of which 232 (55.8%) were male. Of note, 174 (41.8%) were ≥80 years of age. As expected, in-hospital mortality rate was closely associated with age. The overall death rate was 17.8% (74 / 416). In the group ≥80 years of age 23.4% (41/ 174) died, vs. 16.8% (23/137) in the 70-79 year group, and 9.5% (10/105) in the 60-69 year group (p = 0.003). Only one patient (0.2%) died following discharge but within 30 days of the trauma and was considered as in-hospital death.

### Post-discharge survival

The demographic and clinical characteristics of the patients in the post discharge survival category are noted in Table 2. 342 patients were discharged from the hospital and were available for follow up. Of this group, 133 patients (38.9%) were ≥80 years of age. During the follow-up period, 119 patients (34.8%) died (non-survivor group) at a mean follow up of 18.8 months (range: 1.1-66.2 months). 223 patients (65.2%) survived at a mean follow up of 50.2 months (range: 24.8-83.8 months). On univariate analysis, older age was significantly associated with a poor long term outcome (p < 0.0001). Patients who were involved in road traffic collisions, (pedestrians and passengers) were significantly more likely to have a favorable long term outcome compared with those whose mechanism of injury was a fall (p < 0.01). A higher head region AIS was significantly associated with a poorer outcome. Similarly, a low GCS upon admission and the need for intubation at the scene, but not in the ED, were associated with a worse outcome (p < 0.0001, and p < 0.01, respectively). Interestingly, parameters of in-hospital course, including requirement for ICU admission, blood transfusion and in-hospital complications (infectious and non-infectious) did not influence long term outcome (Table 2). Overall LOS was shorter for the survival group but this difference did not reach statistical significance. Ultimate discharge destination was significantly associated with outcome. Patients who were either discharged home or to a rehabilitation facility had a significantly improved long term outcome (p < 0.001) compared to those who were discharged to an ALF.

**Table 2:** Univariate analysis of long term survival

	**Non-survivors**	**Survivors**	**P value**
	**(n = 119)**	**(n = 223)**	
Age (mean ± SD)	80.1 ± 9.64	74.2 ± 9.07	<0.0001
Males (n, %)	66 (55.5)	121 (54.3)	NS
MOI (n, %)			
Fall	93 (78.2)	131 (58.7)	<0.001
MVA car	8 (6.7)	37 (16.6)	0.01
MVA pedestrian	11 (9.2)	46 (20.6)	<0.01
Assault	3 (2.5)	3 (1.3)	NS
Burn	2 (1.7)	2 (0.9)	NS
ISS (mean ± SD)	21.8 ± 7.6	21.8 ± 6.9	NS
Probability of survival (mean ± SD)	78.1 ± 24.65	84.4 ± 19.69	0.01
Head AIS (mean ± SD)	4.21 ± 0.765	3.86 ± 0.944	0.001
GCS upon admission (mean ± SD)	11.85 ± 4.21	13.73 ± 2.89	<0.0001
Intubation (n, %)			
At scene	11 (9.2)	5 (2.2)	<0.01
In ED	8 (6.7)	18 (8.1)	NS
Required operation (n, %)	38 (31.9)	89 (39.9)	NS
LOS (mean ± SD)	20.03 ± 19.51	16.09 ± 16.9	0.05
Admitted to ICU (n, %)	62 (52.1)	111 (49)	NS
Blood transfusion (n, %)	55 (46.2)	104 (46.6)	NS
In-hospital complications (n, %)	23 (19.3)	47 (21.1)	NS
Discharge destination (n, %)			
Rehabilitation	18 (15.1)	66 (29.6)	<0.01
Home	35 (29.4)	112 (50.2)	<0.001
Assistant living facility	65 (54.6)	38 (17.0)	<0.0001
Other hospital	1 (0.8)	7 (3.1)	NS

MOI–mechanism of injury; ED–emergency department; LOS–length of stay; ICU–intensive care unit; SD–standard deviation; MVA–motor vehicle accident; GCS–Glasgow Coma Scale; AIS–abbreviated injury score; ISS–injury severity score; NS–not significant.

### Effect of co-morbidity on survival

The impacts of pre-existing co-morbidities on survival following discharge are noted in Table 3. On univariate analysis, dementia, ischemic heart disease (IHD), diabetes mellitus (DM), and hypertension (HTN) were found to be significantly associated with post discharge death (p < 0.05 for all). Of note, malignancy and COPD failed to impact survival, but the number of patients in these groups was insufficient to draw any conclusions. The mean number of co-morbidities was significantly associated with long-term mortality (p < 0.0001) (Table 3).

**Table 3:** Univariate analysis of the effect of co-morbidities on survival

	**Non-survivors**	**Survivors**	**P value**
	**(n = 119)**	**(n = 223)**	
CRF	11 (9.2)	9 (4.0)	0.05
Anti-coagulant therapy	6 (5.0)	24 (10.8)	0.1
HTN	56 (47.1)	78 (35.0)	0.03
IHD	38 (31.9)	49 (22.0)	0.05
DM	35 (29.4)	39 (17.5)	0.01
COPD	1 (0.8)	2 (0.9)	NS
Dementia	18 (15.1)	1 (0.5)	<0.0001
CVA and/or neurologic disease	20 (16.8)	21 (9.4)	0.05
Malignancy	5 (4.2)	4 (1.8)	NS
≥3 co-morbidities	26 (21.9)	31 (13.9)	0.06
Mean number of co-morbidities	1.6 ± 1.1	1.0 ± 1.2	<0.0001

CRF–chronic renal failure; HTN–hypertension; IHD–ischemic heart disease; DM–diabetes mellitus; COPD–chronic obstructive pulmonary disease; CVA–cerebro-vascular accident.

### Analysis of post-discharge mortality

In order to analyze post-discharge mortality, patients were grouped into an ‘early’ group (mortality < 3 months post-injury) and a ‘late’ group (mortality >3 months post -injury). The pattern of injury, GCS upon arrival, and co-morbidities were not different between the groups. Early post-discharge mortality (≤90 days) occurred in 17 patients (14.3%), while 102 patients (85.7%) died >90 days following discharge (Table 4). Of note, post-discharge mortality was not affected by admission parameters, but by hospital course. Neither age nor mechanisms of injury were found to be risk factors for early post-discharge mortality following injury. Patients who required ICU admission were at increased risk for early death following discharge compared with those who died after a period ≥3 months (14/ 17 [82.4%] vs. 48/102 patients [47.1%], respectively, p < 0.01). Early versus late death was also associated with transfusion of blood products (12 /17 patients [70.6%] vs. 43/102 patients [42.2%], respectively, p = 0.04) and with the development of in-hospital complications (7/17 [41.2%] vs. 16/102 [15.7%], respectively, p = 0.02). ISS was noted to be higher for those who died early, but this difference did not reach statistical significance (mean ISS 25.1 ± 10.7, vs. 21.3 ± 6.9, respectively, p = 0.05). The pattern of injury, GCS upon arrival, and co-morbidities were not different between the groups.

**Table 4:** Univariate analysis of early versus late mortality

	**Early death (<3 months)**	**Late death ( ≥3 months)**	**P value**
	**(n = 17)**	**(n = 102)**	
Age (mean ± SD)	81.1 ± 6.8	79.9 ± 10.0	NS
Males (n, %)	9 (52.9)	57 (55.9)	NS
MOI (n, %)			
Fall	14 (82.4)	79 (77.5)	NS
MVA car	1 (5.9)	7(6.9)	NS
MVA pedestrian	2 (11.8)	8 (7.8)	NS
Other	0 (0)	8 (7.8)	NS
ISS (Median, range)	25 (16-25)	17 (16-25)	0.1
Probability of survival (mean ± SD)	69.9 ± 28.9	79.4 ± 23.6	0.1
Head trauma (n, %)	12 (70.6)	65 (63.7)	NS
GCS upon admission (mean ± SD)	10.9 ± 4.6	12 ± 4.1	NS
Intubation (n, %)			
At scene	2 (11.8)	9 (8.8)	NS
In ED	1 (5.9)	7 (6.9)	NS
Required operation (n, %)	8(47.1)	30 (29.4)	NS
LOS (mean ± SD)	28.8 ± 19.4	18.6 ± 19.2	<0.05
Admitted to ICU (n, %)	14 (82.4)	48 (47.1)	<0.01
Blood transfusion (n, %)	12 (70.6)	43 (42.2)	0.04
In-hospital complications (n, %)	7 (41.2)	16 (15.7)	0.02
Discharge destination (n, %)			
Rehabilitation	2 (11.8)	16 (15.7)	NS
Home	1 (5.9)	34 (33.3)	0.02
Assistant living facility	14 (82.4)	51 (50.0)	0.02
Other hospital	0 (0.0)	1 (1.0)	NS

NS–not significant; MOI–mechanism of injury; MVA–motor vehicle accidents; ED–Emergency Department; ICU–intensive care unit. Data shown as number (and percentage) and mean (±SD).

### Predictors of long-term survival

**Figure 1:**
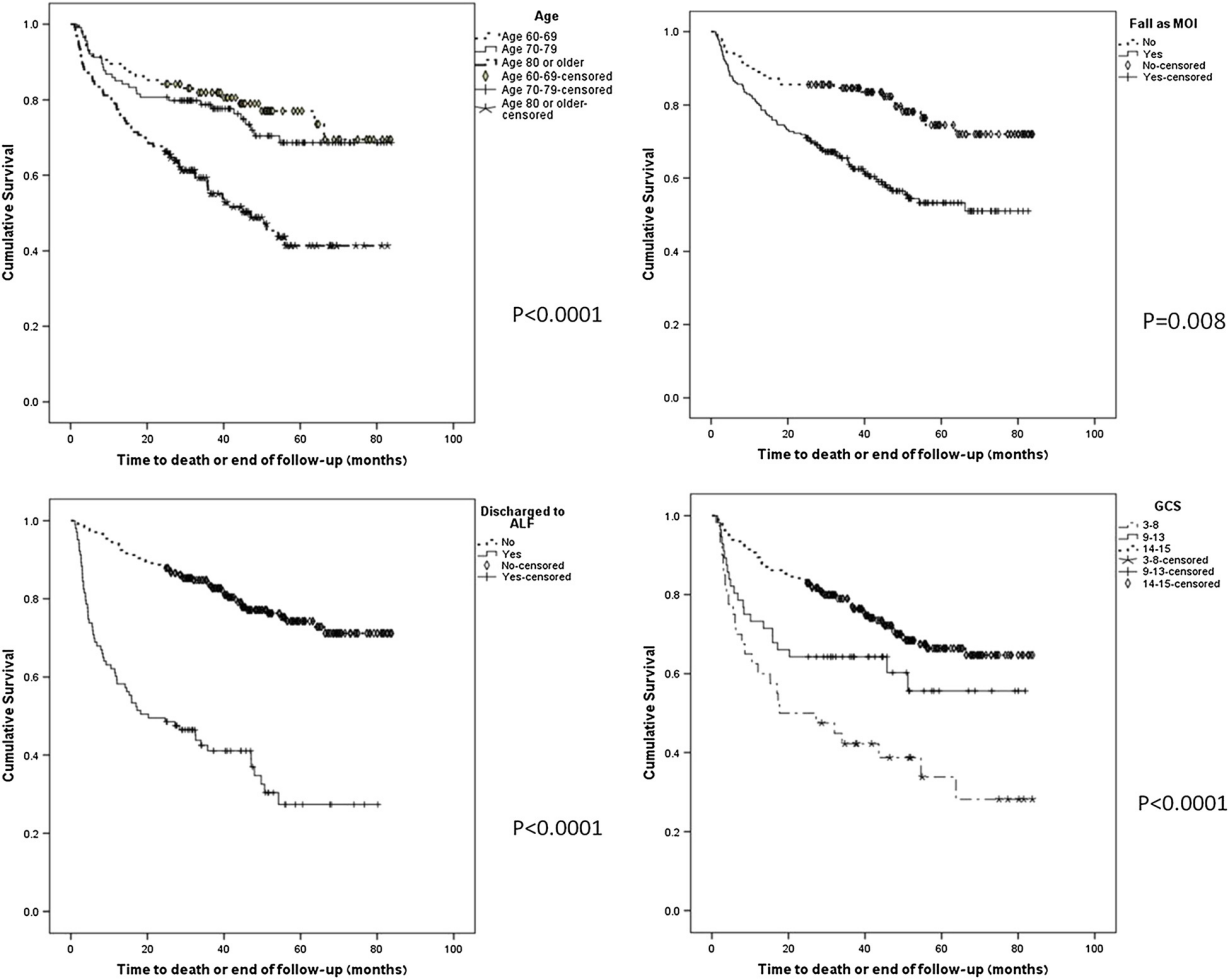
Cox regression model for parameters predicting early post discharge death: age >80; fall as a mechanism of injury; discharge to assisted living facility (ALF); low GCS on arrival to emergency department.

Univariate survival curves demonstrated that age, mechanism of injury, GCS upon admission and discharge destination were significantly associated with long-term survival (Figure 1). Multivariate analysis was performed to analyze those factors predictive of survival. Parameters which were found to be significant on univariate analysis were entered into a forward stepwise Cox regression model. As noted age, fall as mechanism of injury, GCS and renal failure upon admission and discharge destination were found to be predictors of long term survival (Table 5).

**Table 5:** Predictors of long term survival in severely injured elderly trauma patients

	**Adjusted hazard ratio**	**95% confidence interval**	**P value**
Age	1.044	1.022-1.065	<0.0001
Fall as mechanism of injury	1.90	1.181-3.057	<0.01
Low GCS in ED	0.883	0.845-0.924	<0.0001
Creatinine in ED	1.003	1.000-1.005	0.03
Discharge to ALF	0.315	0.214-0.463	<0.0001

GCS–Glasgow coma scale; ED–emergency department; ALF–assisted living facility.

## Discussion

The major finding of this study is that in the elderly population following severe trauma, long term survival can be predicted based on the pre-hospital parameters of age, mechanism of injury, and GCS on admission. In contrast, parameters in hospital care, including blood transfusion, requirement for ICU admission, surgical procedures and complications did not predict long term survival in this elderly group.

There is a paucity of data describing the long term outcome of the injured geriatric patient, accordingly, this was a primary objective of our study. Contrary to what is often assumed, we have demonstrated that long term survival subsequent to a severe trauma in the elderly population is not uncommon, for we noted that almost two-thirds of elderly patients who were discharged from the hospital were alive at a mean follow up of over 4 years.

Previous reports have analyzed the course and in-hospital outcome of elderly patients following trauma [4,11,12]. A mature trauma system performance could be assessed by the percent of severely injured patients who are discharged from the trauma center. For example, Florida trauma system analysis over a 15 year period showed significant increase in both the number of elderly injured and the severity of injury [13]. Others [14] stressed the importance of triage of the severely injured elderly patients to designated trauma centers. This resulted in significantly higher overall discharge when compared to non-trauma centers.

Not surprisingly, and in concert with others [4,15] our data demonstrated that chronological age is a predictor of post-discharge mortality. The post-discharge survival of patients ≥ 80 years is significantly worse compared to their younger counterparts. These intuitive findings could not be explained by the ISS, which was not different between the age groups. Although age related co-morbidities likely contribute to long term survival, we were surprised to note that age, rather than co-morbidities and ISS, was an independent predictor of death, particularly in the ≥80 age group.

It has been noted that in the elderly population, multi-system trauma from falls predominant with increasing age, with a corresponding decreasing frequency of motor vehicular and pedestrian related injuries [5]. Similarly, we noted that falls were the most common mechanism of injury and were associated with poor long term outcome. It has been suggested that a senior’s propensity to fall may indicate poor functional capacity and higher mortality risk in this population [16].

Various studies confirm that pre-existing co-morbidities significantly increase the risk of mortality following blunt trauma in geriatric patients [17-20]. The association between DM and early death in the elderly population has been previously noted for general in-hospital admissions [21,22]. Similarly, we noted that the most common pre-existing co-morbidities in our population were HTN, followed by IHD and DM. On univariate analysis these conditions and dementia were associated with poor long term survival. However, on multivariate analysis none of these co-morbidities predicted long term survival. Interestingly, the mean number of co-morbidities was also associated with poor long term outcome.

Traumatic brain injury in geriatric patients has been recognized to result in a worse outcome when compared to younger counterparts, with a low admission GCS commonly recognized as a poor prognostic indicator [23]. Others [24] have argued that perhaps poor overall condition, rather than head injury, per se, determines outcome. We noted that a low GCS, and not head AIS, was found to be an independent predictor of post-discharge mortality. It may be argued that the general condition of the patient, and not the exact type of head injury, is what determines long term outcome [24].

Our finding that more than half of patients in our study required ICU admission (173 patients, 50.6%) and over a third of that group required an operation confirms the fact that considerable acute care resources were utilized for the treatment of these seriously injured elderly patients.

Demographics, pre-hospital and admission parameters could not predict the likelihood of early post-discharge death (within 3 months of injury). However, in-hospital course including the need for ICU admission, blood transfusion and in-hospital complications were found to be associated with early (<3 month) post-discharge mortality. Thus, our data suggest that the characteristics of early post-discharge death may be more similar to in-hospital death than to death during long term follow up.

While our study does not contain data concerning the cost of trauma care in this population, the financial burden of end of life care has been well described [25]. Accordingly, one might surmise that recognition of parameters that aid in predicting long term survival in these patients would avert the allocation of limited resources and funds on patients with a predicted poor outcome. Currently, in our country and in our institution, there are no limitations in hospital resource allocation for injured elderly patients, although continued concerns world-wide for the costs of care could lead to such limitations. Accordingly, we and others [13,14] believe that increased attention to the growing burden of geriatric trauma care is imperative for future trauma system design, performance improvement, and resource allocation in an effort to improve outcomes in this group.

Legner et al [26] demonstrated a 3.5 times greater mortality at 1 year for patients ≥65 years of age undergoing abdomino-pelvic surgery discharged to a skilled nursing facility compared with those discharged home. Not surprisingly, we found that discharge to an ALF (27% of patients) had a negative impact on long term outcome. This can be explained by the significant differences in physical therapy and occupational therapy options available for patients in rehabilitation programs compared with patients at ALF. Selection bias of patients in a poorer overall condition to ALF could also explain these findings.

There are a number of significant strengths and limitations of this study. Inclusion criteria were ISS >15 thus making this cohort of patients appropriate for the study of long term survival. We excluded patients who died in the hospital from the analysis of delayed long term mortality because the acute mortality from major trauma is determined largely by the severity of the initial injury. This study design allowed us to potentially separate the effects of the initial injury, but rather to use the initial data of patient admission to predict long term outcome.

The major limitation of this study is related to retrospective data analysis. In our trauma registry co-morbidities are listed by reviewing previous discharge letters with the incumbent limitations of such data. Finally, data on pre-injury living status for the 148 patients who returned home is not available, and therefore, we cannot draw any definitive conclusions regarding the home status of this group.

In conclusion, we have shown that clinical and demographic factors are associated with long term, post-discharge outcome following severe trauma in geriatric patients, and we noted that almost 2/3 of elderly patients injured following a trauma were discharged from the hospital with a favorable long term outcome. We noted that common demographic and clinical parameters, including age ≥ 80, lower GCS upon arrival and fall as the mechanism of injury are clear predictors of a poor long term outcome for severely injured geriatric trauma patients.

Although most studies commonly evaluate in hospital, < 30 day mortality, our findings expands our understanding of factors contributing towards long term post-discharge survival. Given the substantial and increasing burden of the elderly sustaining traumatic injury, our findings underscore the importance of additional research to further identify risks and prognostic factors to improve our trauma care and performance improvement, in order to ultimately impact survival in the injured elderly patient. The role of a geriatric consultation service could be crucial in their care and play an important role in the framework of a multi-disciplinary team.

## Competing interests

All authors declare that they have no competing interests.

## Authors’ contributions

MB–literature search, study design, data collection, data analysis, data interpretation, writing, critical revision. JLK–study design, data interpretation, writing, critical revision. DW–data analysis, data interpretation, writing, critical revision. DK–data collection, data analysis. TBA–data analysis, data interpretation. GA–literature search, study design, data collection, data analysis, data interpretation, writing, critical revision. All authors read and approved the final manuscript.
